# Bilateral versus unilateral orchidopexy: IVF/ICSI-ET outcomes

**DOI:** 10.3389/fendo.2024.1294884

**Published:** 2024-02-01

**Authors:** Lijuan Fan, Lin Shi, Shan Liu, Zhou Zhang, Juanzi Shi

**Affiliations:** ^1^ Assisted Reproduction Center, Northwest Women’s and Children’s Hospital, Xi’an, China; ^2^ Department of Immunology and Microbiology, Xi’an Jiaotong University College of Medicine, Xi’an, China

**Keywords:** cryptorchidism, orchidopexy, *in vitro* fertilization, intracytoplasmic sperm injection, live birth

## Abstract

**Introduction:**

Cryptorchidism is a common genital disorder. Approximately 20% of azoospermic or infertile men reported having histories of cryptorchidism. Bilateral cryptorchidism may have been more condemned than unilateral cryptorchidism. Early treatment by orchidopexy is the definitive procedure for cryptorchid patients with cryptorchidism. However, fertility potency after orchidopexy may be adversely affected and assisted reproduction techniques will be required for infertile patients.

**Objective:**

To compare the reproductive outcomes between unilateral and bilateral orchidopexy groups.

**Methods:**

A retrospective cohort study at a tertiary hospital, including a total of 99 infertile men who underwent orchidopexy to treat cryptorchidism and subsequently underwent their first IVF/ICSI-ET cycle. Men were grouped according to the laterality of their cryptorchidism and orchidopexy surgeries they received. Fertilization rate and live birth rate were chosen as parameters for evaluating outcomes.

**Results:**

The sperm concentration and viability were significantly higher in unilateral orchidopexy group than in bilateral orchidopexy group (28.09 ± 27.99 vs 7.99 ± 14.68, *P*=0.001; 33.34 ± 22.52 vs 11.95 ± 17.85, *P*=0.001). Unilateral orchidopexy group showed lower demand for ICSI (66.07% vs 95.35%, *P*<0.001). Interestingly, both groups exhibited similar rates of fertilization, clinical pregnancy, live birth and birth defect. Boy birth ratio was lower in bilateral orchidopexy group as compared to unilateral orchidopexy group (27.27% vs 58.62%, *P*=0.026).

**Conclusion:**

A history of bilateral orchidopexy surgery correlates with a worsened sperm parameter and a higher demand for ICSI as compared to patients with history of unilateral orchidopexy. However, this does not influence the final live birth rate.

## Introduction

Cryptorchidism refers to the absence of one or both testes from the scrotum, which could result in impaired fertility (1). Nearly 1% of male children are affected by cryptorchidism and one-third of these patients have bilaterally cryptorchid testes. At the same time, cryptorchidism is one of the most frequent causes of non-obstructive azoospermia (NOA) in adulthood (2, 3). Approximately 20% of azoospermic or infertile men reported histories of cryptorchidism (4). The etiology of the cryptorchidism is considered to be multifactorial, including endocrinal, environmental, genetic, anatomical and mechanical factors. Hence, it is generally considered a complex disease (5). Early treatment by orchidopexy is the definitive procedure for cryptorchid patients with undescended testis. However, fertility potency after orchidopexy may be adversely affected and assisted reproduction techniques will be required for infertile patients.

A study (1) was performed to compare the testicular sperm extraction (TESE) Intracytoplasmic sperm injection-embryo transfer (ICSI-ET) outcomes between bilateral and unilateral cryptorchidism in NOA patients. Although 90.36% of their study population have undergone orchidopexy to improve their conditions at around 9 to 10 years old, the fertilization rates of bilateral and unilateral cryptorchidism group were only at 46.8% and 46.5% respectively, which were slightly below the average ICSI fertilization rate. Bilateral cryptorchidism may have been more condemned than unilateral cryptorchidism (6). Barbotin found that patients of bilateral cryptorchidism had lower implantation rate. The study especially focused on cryptorchidism patients with complications from NOA. Apart from the mentioned study, no other study has attempted to evaluate the IVF/ICSI-ET outcomes in infertile men who had undergone orchidopexy. The purpose of our present study was to evaluate the IVF/ICSI-ET outcomes of infertile patients of unilateral and bilateral cryptorchidism.

## Materials and methods

### Study design and patients

This study is a retrospective cohort analysis and our data were obtained from the electronic medical record system of Northwest Women’s and Children’s Hospital from January 2014 and January 2022. Patients were enrolled if they met the following criteria: [1] men who had orchidopexy to treat cryptorchidism [2] in the first cycle of IVF/ICSI-ET [3] wives’ age were less than 42 years old. The exclusion criteria included the following: men who [1] had contralateral testicle resection, absence, hernia or hydrocele; [2] adopt donor sperm; [3] PGT cycles; wife diagnosed [4] with uterine pathology (including unicornuate uterus, duplex uterus, rudimentary uterus, intrauterine adhesions and septate uterus); or [5] untreated hydrosalpinx.

All the patients received follow up till a year after embryo transfer. The study was conducted with the approval of the hospital ethics committee (number 2021002).

### Analysis and evaluation before IVF/ICSI-ET

Semen analysis, chromosome karyotype and Y chromosome deletion were presented in each patient. Semen analysis was performed with reference to the WHO guidelines. Chromosomal karyotype was detected using the G-banding method in accordance to the specifications of ISCN2009. Y chromosome deletion was analyzed using STSs, (sequence tagged sites were listed in **Supplementary Table 1**). Insemination method was selected according to the sperm count after sperm preparation. When the cell count was more than 5×10^6^ motile sperm/mL, IVF insemination method was selected. And when the cell count was less than that, ICSI insemination method was selected. Nonobstructive azoospermia was defined as no sperm observed by microscopic examinations after centrifugal precipitation in at least three routine semen examinations, excluding those with obstructive azoospermia.

### Controlled ovarian hyperstimulation

Controlled ovarian hyperstimulation (COH) protocols included GnRH agonist protocol and GnRH antagonist protocol. The chose of ovarian stimulation protocol was based on antra follicle counts, regularity of menstruation and doctor’s preferences. The initialing does of gonadotropin varies from 100 IU to 300 IU, which was mainly determined on the number of antra follicle counts. HCG injection (Ovidrel, Merck Serono, Italy) was administered to patients when leading follicle reached 18 mm, and oocyte retrieval was performed 36 hours after HCG injection.

### Fertilization

Testicular sperm aspiration (TESA) and microdissection TESE were recommended to patients with NOA. TESA was performed in the following steps: after lidocaine was injected around the testicle, biopsy needle was passed into the testicle to collect testicular tissue. Testicular tissue will be flushed into a small plastic petri dish which was then transferred to IVF laboratory for fertilization. Microdissection TESE was performed as previously described by Guo (7). After a longitudinal incision was made along the median raphe, the testicle was delivered through the tunica vaginalis. Albuginea incision and microdissection of seminiferous tubules were performed under microscope. The most morphologically normal motile spermatozoon was selected for ICSI.

ICSI was performed on a heated platform. Oocytes were placed individually into droplets of IVF solution covered under warm mineral oil. Sperm were placed in a central droplet of polyvinylpyrrolidone solution, and the procedure was performed on the heated stage of an inverted microscope. Embryologist injected the sperm into metaphase II oocyte using direct penetration technique.

Conventional IVF fertilization was performed 2 to 2.5 h after oocyte retrieval. Each oocyte was incubated with approximately 40,000 sperm and fertilization is allowed to occur naturally. Short-term fertilization was applied and the cumulus granule cells were peeled off after 4 hours of fertilization. Fertilization was assessed 16 h to 18 h following routine IVF or 12 h after ICSI. The presence of two pro-nuclei indicated normal fertilization. ART technique process and culture medium of the two groups were identical.

### Embryo evaluation and transfer

A morphologic score of cleavage-stage embryo was given based on the number of blastomeres, the homogeneous degree of blastomeres, and the degree of cytoplasmic fragmentation (8). If a couple has two or more good quality cleavage-stage embryos on day 3 of embryo culture, an embryo will be selected and cultured to blastocyst stage. Day 5 and Day 6 blastocyst evaluation was performed according to Gardner Grade Standard (9).

The number of embryos transfer was mainly based on the quality of embryo and women age. Elective single blastocyst transfer (eSBT) was recommended to young woman who had good quality blastocysts. The embryo with best grade was selected for transfer. Among women above 35 years old or who did not have good quality embryos, double ET was suggested instead, according to our practices (10). Luteal support began from the day of embryo transfer, and 90 mg of Crinone (EMD Serono, UK) and 20 mg of oral progesterone (Abbott, Netherlands) were provided to the patients on a daily basis.

### Pregnancy Detection

Serum β-hCG test was performed 2 weeks after ET to test for pregnancy. Luteal support would continue to be provided if the β-hCG level was >50 IU/L on the 2nd week after ET. Clinical pregnancy was confirmed when the existence of an embryo with cardiac activity was detected by ultrasonographic visualization around 5 weeks after ET.

### Definition of reproductive outcomes

Our primary outcomes were focused on fertilization rate and live birth rate. The fertilization rate was defined as the number of normally fertilized oocytes divided by the number of oocytes inseminated. Live birth was the delivery of a living infant beyond 22 weeks of gestation. Live birth rate was defined as the number of live birth cycles divided by number of embryo transfer cycles.

The secondary outcomes included clinical pregnancy rate, miscarriage rate, preterm birth rate, birth defect rate and sex ratio of neonates. Clinical pregnancy rate was defined as the number of clinical pregnancy cycles expressed divided by embryo transfer cycles. Miscarriage rate was defined as the number of spontaneous pregnant loss before the 22nd week, divided by the number of clinical pregnant. Preterm birth was defined as a birth that took place between the 22nd and 37th week of gestational age. Birth defects were determined based on the tenth edition of International Classification of Diseases Clinical Modification Code.

### Statistical analysis

All statistical analyses were performed using SPSS version 19.0 (SPSS Inc, USA). Normally distributed continuous variables were compared using Student’s t-test. Rates were compared using the χ^2^ test. Binary logistic regression models were used to calculate odds ratios and 95% confidence intervals of reproductive outcomes, and to evaluate the effect of potential confounders (11). Differences were considered statistically significant at *P*<0.05.

## Results

**Figure 1:**
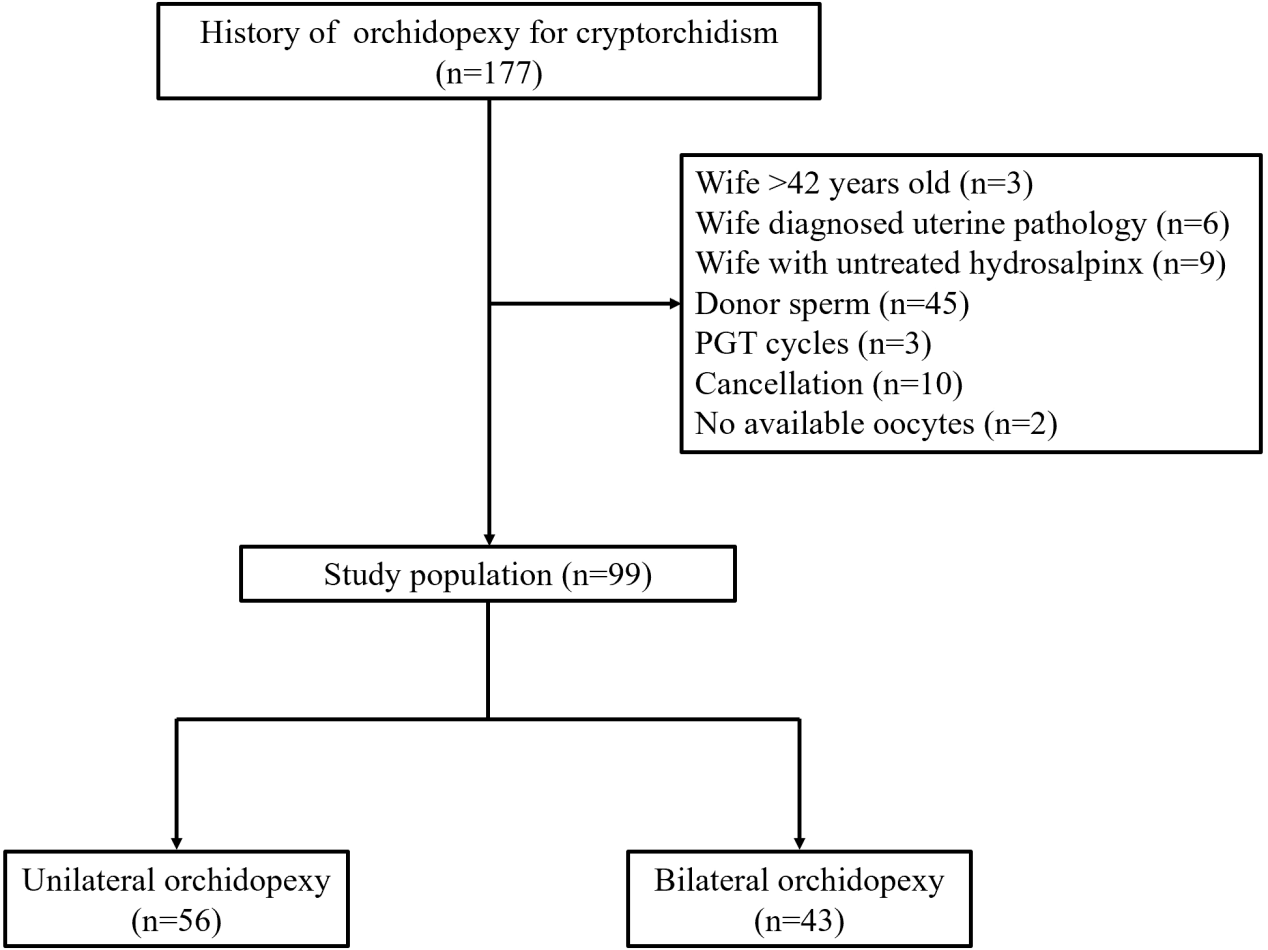
A flow chart of the studied population.

The flowchart of enrolled patients is shown in **Figure 1**. For the entire study, 99 male patients were included eventually, in which 56 had history of unilateral orchidopexy and 43 had history of bilateral orchidopexy. The baseline profiles of enrolled patients are summarized in **Table 1**. There was no significant difference between the two groups in couples’ age, BMI, infertility duration and type (*P*>0.05). Notably, in the cause of infertility, the proportion of male factor was significantly higher in bilateral orchidopexy group when compared with the unilateral orchidopexy group (93.02% vs 51.79%, *P*<0.001). The proportion of female factor (32.14% vs 4.56%, *P*=0.001) and couple factor (16.07% vs 2.33%, *P*=0.040) were significantly higher in unilateral orchidopexy group. A case of Klinefelter’s syndrome (KS) was found in each group. Two patients (2.74%) from the bilateral orchidopexy group were found with Y chromosome AZFc microdeletion. Antral follicle count, basal FSH and LH were similar in both groups (each at *P*>0.05).

**Table 1:** Baseline characteristics of patients.

characters	Unilateral orchidopexy group n=56	Bilateral orchidopexy group n=43	*P* value
Male age (y)	31.35 ± 4.00	30.60 ± 5.07	0.409
Male BMI (Kg/m^2^)	24.60 ± 3.80	25.40 ± 3.50	0.291
Infertility duration	3.49 ± 2.64	3.44 ± 2.24	0.918
Infertility type (%)			
Primary	82.14 (46/56)	88.37 (38/43)	0.573
Secondary	17.86 (10/56)	11.63 (5/43)	
Cause of infertility (%)			
Male factor	51.79 (29/56)	93.02 (40/43)	<0.001
Female factor	32.14 (18/56)	4.65 (2/43)	0.001
Couple factor	16.07 (9/56)	2.33 (1/43)	0.040
Male Abnormal karyotype (%)	1.79 (1/56)	2.33 (1/43)	>0.999
Y chromosome deletion (%)	0.00 (0/56)	4.65 (2/43)	0.186
Female age (y)	29.68 ± 4.17	28.60 ± 3.36	0.167
Female BMI (Kg/m^2^)	22.23 ± 3.51	21.89 ± 3.34	0.628
AFC	12.54 ± 5.36	12.95 ± 6.02	0.723
Basal FSH (IU /L)	7.15 ± 2.92	7.19 ± 3.06	0.961
Basal LH (IU/L)	4.26 ± 1.81	4.84 ± 1.74	0.116

Data are expressed as mean ± SD or rate (positive number/total number); BMI, body mass index; AFC, antral follicle count at baseline; FSH, follicle-stimulating hormone; LH, luteinizing hormone.

**Table 2** shows the semen analysis, COH and fertilization outcomes. The occurrences of azoospermia were 33.93% and 43.49% in unilateral and bilateral orchidopexy group, respectively (*P*=0.066). Among patients without azoospermia, sperm concentration and sperm viability of the unilateral orchidopexy group were significantly higher than that of the bilateral orchidopexy group (28.09 ± 27.99 vs 7.99 ± 14.68, *P*=0.001; 33.34 ± 22.52 vs 11.95 ± 17.85, *P*=0.001). In the controlled ovarian hyperstimulation portion, two groups were similar in terms of gonadotropin (Gn) duration and Gn dosage (each at *P*>0.05). The average number of oocytes retrieved in unilateral orchidopexy group was significantly lesser than the bilateral orchidopexy group (10.19 ± 5.27 vs 13.83 ± 7.08, *P*=0.004). The proportion of IVF fertilization was significantly higher in unilateral orchidopexy group, compared with the bilateral orchidopexy group (33.93% vs 4.65%, *P*<0.001). Unilateral orchidopexy group showed lower demand for ICSI (66.07% vs 95.35%, *P*<0.001). In unilateral and bilateral orchidopexy group, 35.71% and 48.84% of the participants obtained sperm through testicular surgery, respectively (*P*=0.220). There were no significant differences between the two groups with regards to the fertilization rate and blastocyst formation rate (each at *P*>0.05). Three cases from the unilateral orchidopexy group and two cases from the bilateral orchidopexy group failed to fertilize. In addition, three cases from the bilateral orchidopexy group were unsuccessful at embryo culture. The abovementioned eight cases had no embryos to transfer.

**Table 2:** Semen analysis, COH and fertilization outcomes.

	Unilateral orchidopexy group n=56	Bilateral orchidopexy group n=43	*P* Value
Azoospermia (%)	33.93 (19/56)	53.49 (23/43)	0.066
Sperm concentration (10^6^/ml)	28.09 ± 27.99	7.99 ± 14.68	0.001
Sperm viability (%)	33.34 ± 22.52	11.95 ± 17.85	0.001
Gn duration (d)	10.44 ± 2.24	10.66 ± 2.07	0.621
Gn dosage (IU)	2265.35 ± 867.35	2115.85 ± 798.85	0.387
NO. oocyte retrieved (n)	10.19 ± 5.27	13.83 ± 7.08	0.004
Fertilization type (%)			
IVF	33.93 (19/56)	4.65 (2/43)	<0.001
ICSI	66.07 (37/56)	95.35 (41/43)	
Testicular surgery ICSI	35.71 (20/56)	48.84 (21/43)	0.220
Fertilization rate (%)	62.77 ± 22.06	54.60 ± 27.03	0.113
Blastocyst formation rate (%)	32.68 (67/205)	28.75 (46/160)	0.427

Data are expressed as mean ± SD or rate (positive number/total number); COH, controlled ovarian hyperstimulation; IVF, *in vitro* fertilization; ICSI, intracytoplasmic sperm injection.

There were no significant differences between the two groups in terms of the fresh/frozen-thawed embryo transfer ratio, endometrial thickness, number of embryos transferred, good quality embryo transfer ratio and type of embryo transferred (each at *P*>0.05) (**Table 3**). Also, clinical pregnancy rate, implantation rate, multiple pregnancy rate, miscarriage rate, preterm birth rate and live birth rate of the two groups were similar (each at *P*>0.05).

**Table 3:** Outcomes of embryo transfer.

	Unilateral orchidopexy group n=53	Bilateral orchidopexy group n=38	*P* Value
Transfer cycle (%)			
Fresh cycle	69.81 (37/53)	68.42 (26/38)	0.887
Frozen-thawed cycle	30.19 (16/53)	31.58 (12/38)	
Endometrial thickness (mm)	11.15 ± 2.58	11.32 ± 2.66	0.764
NO. of embryos transferred (n)	1.53 ± 0.50	1.54 ± 0.51	0.974
Good quality embryos transferred ratio (%)	40.74 ± 45.63	36.49 ± 43.54	0.657
Type of embryos transferred (%)			
Cleavage-stage embryo	58.49 (31/53)	57.89 (22/38)	0.955
Blastocyst-stage embryo	41.51 (22/53)	42.11 (16/38)	
Clinical pregnancy rate (%)	66.04 (35/53)	57.89 (22/38)	0.428
Implantation rate (%)	52.44 (43/82)	44.83 (26/58)	0.375
Multiple pregnancy rate (%)	8.57 (3/35)	13.64 (3/22)	0.667
Miscarriage rate (%)	22.86 (8/35)	18.18 (4/22)	0.750
Preterm birth rate (%)	0.00 (0/35)	9.09 (2/22)	0.1450
Live birth rate (%)	49.06 (26/53)	52.63 (20/38)	0.120
Boy ratio (%)	58.62 (17/29)	27.27 (6/22)	0.026
Birth defect (%)	2.86 (1/35)	0.00 (0/22)	0.424

Data are expressed as mean ± SD or rate (positive number/total number).

To further investigate the rollover effect of cryptorchidism laterality on offspring, we compared the sex ratio and birth defects between the groups. Boy birth ratio of bilateral orchidopexy group was 27.27%, which was significantly lower than the unilateral orchidopexy group (58.62%, *P*=0.026). Both patients with KS in our study had unsuccessful pregnancies. The patient with KS in the unilateral orchidopexy group experienced embryo implantation failure, while the other patient in the bilateral orchidopexy group failed during embryo culture. One newborn from the unilateral orchidopexy group was diagnosed with congenital heart disease. The birth defect ratios of the two groups were similar (*P*>0.05).

Univariate analysis of the live birth rate is shown in **Table 4**. Infertility duration (OR 0.626; 95%CI 0.394, 0.994) was considered a risk factor for live birth (*P*=0.047). Blastocyst-stage embryo transfer (OR 3.849; 95%CI 1.171, 12.651) was a profitable factor for live birth compared to cleavage-stage embryo transfer (*P* =0.026). History of unilateral or bilateral orchidopexy, age, BMI, basal FSH, basal LH, sperm concentration, motility, number of oocytes retrieved, number of embryos transferred, number of good quality embryos transferred and endometrial thickness did not affect live birth rate (*P*>0.05).

**Table 4:** Univariate analysis for live birth rate.

Covariate	OR (95% CI)	*P* Value
Female age	0.899 (0.644,1.255)	0.531
Female BMI	0.961 (0788,1.173)	0.698
Infertility duration	0.626 (0.394,0.994)	0.047
Male age	1.137 (0.871,1.485)	0.346
Male BMI	0.991 (0.797,1.233)	0.937
Basal FSH	1.013 (0.859,1.196)	0.874
Basal LH	0.916 (0.717,1.171)	0.485
History of orchidopexy		
Unilateral orchidopexy	Reference	
Bilateral orchidopexy	2.614 (0.499,13.691)	0.256
Sperm concentration	1.013 (0.982,1.046)	0.420
Sperm motility	1.005 (0.969,1.043)	0.771
No. of oocyte retrieved (n)	1.019 (0.931,1.116)	0.681
No. embryos transferred	1.071 (0.354,3.235)	0.904
No. good quality embryo transferred (n)	1.005 (0.994,1.016)	0.384
Type of embryos transferred		
Cleavage-stage embryo	Reference	
Blastocyst-stage embryo	3.849 (1.171,12.651)	0.026
Endometrial thickness (mm)	1.184 (0.982,1.428)	0.077

BMI, body mass index; FSH, follicle-stimulating hormone; LH, luteinizing hormone.

A logistic regression model was then used to assess the association between orchidopexy laterality and live birth rate (**Table 5**). After adjusting for the age of female patients, infertility duration, number of oocytes retrieved and type of embryo transferred, we found orchidopexy laterality had no association with live birth rate (OR 1.274; 95% CI 0.515, 3.151; *P*=0.601).

**Table 5:** Relationship between laterality of cryptorchidism and main outcomes.

	Crude Model		Adjusted Model	
	OR (95% CI)	*P* Value	OR (95% CI)	*P* Value
Unilateral orchidopexy	Reference		Reference	
Bilateral orchidopexy	1.363 (0.591,3.147)	0.468	1.274 (0.515, 3.151)	0.601

## Discussion

The present study summarized the IVF/ICSI-ET outcomes in infertile men with a history of cryptorchidism. We set relatively strict inclusion criteria where only patients who have undergone their first IVF/ICSI-ET treatment could be included. The data of each eligible cryptorchidism patient were included in our study only once, which makes our results perhaps more convincing than previous studies that took ET cycles as study units.

Previous studies (1) had a larger sample size but indicated lower fertilization rate (46.8% and 46.5%) after TESE in bilateral and unilateral cryptorchidism-complicated NOA when compared to our present study (54.60% and 62.77%). Barbotin et al. reported that the implantation rates of bilateral and unilateral cryptorchidism in NOA were 11.9% and 25.5% respectively. In our present study, the implantation rates of bilateral and unilateral cryptorchidism group were 44.83% and 52.44%. The higher fertilization rate was attributed to the difference in our study population. Barbotin et al. included patients who were diagnosed with cryptorchidism in NOA, and all their participants underwent TESE for sperm retrieval. In our present study, we enrolled only patients who were diagnosed with cryptorchidism, where 57.58% of our patients were not azoospermia. Our higher implantation rate was mainly due to higher good quality embryo transfer (36.49% – 40.74%) as compared to Barbotin’s study (25% – 28.7%). Besides, blastocyst-stage embryo transfer accounts for more than 40% of our embryo transfer cycles. Our results showed similar fertilization rate, clinical pregnancy rate and live birth rate in men with a history of either unilateral or bilateral cryptorchidism. Furthermore, the clinical pregnancy rate and live birth rate of our study groups were similar with the rates of the general infertility population (12, 13), suggesting that patients with a history of cryptorchidism can also achieve similar live birth rate as the general population, as long as sperm is available for IVF/ICSI procedures.

At the start, six patients were determined to have KS following chromosome karyotype analysis. The incidence rate of KS in our study population was 3.85% (6/156), which was higher than the estimated prevalence of 1 in 600 live births in males (14). Cryptorchidism is one of the most frequently occurred clinical signs in patients with KS. The prevalence of cryptorchidism in KS patients was 55.5% (15). One of our primary focuses was fertilization rate; as such four patients of KS were excluded from the study as they used donors’ sperm. The remaining two KS patients abandoned PGT and chose ICSI instead. One of them failed during embryo culture while the other one was unsuccessful at embryo implantation.

Although sperm retrieval rate was similar for patients of bilateral or unilateral cryptorchidism (1), unilateral orchidopexy usually has lesser impact on sperm cells (16, 17). Our results are in good agreement with other reports from previous studies, where we similarly observed a lower sperm density and viability in bilateral orchidopexy group as compared to unilateral orchidopexy group. Patients from bilateral orchidopexy group also had a higher demand for ICSI.

Birth defect is another concern for patients with cryptorchidism. We followed up with patients for one year after embryo transfer. Only one infant from the unilateral orchidopexy group was diagnosed with esotropia. There is still no evidence or study establishing the genetic relationship, although the influence of cryptorchidism on genes has been proven in animals (18).

Women’s age, embryo culture condition, fertilization method, and the number of blastomeres at cleavage stage were reported key factors which are associated with blastocyst formation. The blastocyst formation rate in our center was ~60% (19). However, the blastocyst formation rates of both bilateral and unilateral cryptorchidism were nearly half of our normal blastocyst formation rate. Blastocyst formation rate of patients with history of cryptorchidism has not been described before. A larger sample size studies would be needed to prove the association between cryptorchidism and blastocyst formation.

Our retrospective study found that infertility duration increased the risk of live birth in patients with history of orchidopexy. Moreover, blastocyst-stage ET exhibited a higher live birth rate than cleavage-stage ET. Expanding on embryonic culture may increase the likelihood of selecting embryos with better quality for transfer. Our study suggested that blastocyst-stage ET is a good strategy for patients with history of orchidopexy.

Our present work has two notable strengths. First, the assisted reproduction data of our patients are complete. All the patients received follow up till a year after ET. Second, our study results are more convincing than previous studies. We set relatively strict inclusion criteria where only patients who underwent their first IVF/ICSI-ET treatment could be included. This reduces the bias caused by repeated enrolment of the same patient.

This study was limited in that the data source captured only patients from a single medical center, which could lead to selection bias, small sample size and limited external validity to the findings.

## Conclusion

A history of bilateral orchidopexy surgery correlates with a worsened sperm parameter and a higher demand for ICSI as compared to unilateral orchidopexy. Fortunately, this does not influence the final live birth rate. The laterality of cryptorchidism and orchidopexy surgeries were not associated with reproductive outcomes.

## Data availability statement

The original contributions presented in the study are included in the article/**Supplementary Material**. Further inquiries can be directed to the corresponding author.

## Ethics statement

The studies involving humans were approved by Northwest Women’s and Children’s Hospital. The studies were conducted in accordance with the local legislation and institutional requirements. The participants provided their written informed consent to participate in this study.

## Author contributions

LF: Investigation, Methodology, Writing – original draft. LS: Data curation, Formal analysis, Funding acquisition, Writing – original draft. ZZ: Project administration, Writing – review & editing. JS: Project administration, Writing – review & editing. SL: Writing – review & editing.
