# Supplementary material for: Bilateral versus unilateral orchidopexy: IVF/ICSI-ET outcomes
Source: Front Endocrinol (Lausanne). 2024 Feb 1;15:1294884. doi: 10.3389/fendo.2024.1294884 (PMC10867241; doi:10.3389/fendo.2024.1294884)
Supplement: Supplementary file 1 [file Table_1.docx]

Supplemental table 1. The sequence tag sites used for Y chromosome deletion

| AZFa | AZFb | AZFc |
| --- | --- | --- |
| sY84 | sY127 | sY254 |
| sY86 | sY134 | sY255 |
| sY81 | sY121 | sY145 |
| sY85 | sY124 | sY152 |
| sY182 | sY128 | sY153 |
| sY608 | sY130 | sY157 |
| sY741 | sY133 | sY239 |
| sY1323 | sY117 | sY242 |
| sY2323 | sY850 | sY802 |
|  | sY1002 | sY856 |
|  | sY2597 | sY1191 |
|  | sY2832 | sY2713 |
|  | sY2833 | sY2900 |
|  |  | sY2928 |

*AZF* azoospermia factor
